# Healthcare resource use associated with the diagnosis of transthyretin amyloidosis cardiomyopathy

**DOI:** 10.1002/hsr2.466

**Published:** 2022-01-06

**Authors:** Clint Asher, Andrew Guilder, Gherardo Finocchiaro, Gerry Carr‐White, Yael Rodríguez‐Guadarrama

**Affiliations:** ^1^ School of Biomedical Engineering and Imaging Sciences Rayne Institute, King's College London London UK; ^2^ Department of Cardiology Guy's and St Thomas' NHS Foundation Trust London UK; ^3^ Care Redesign Improvement and Innovation System Guy's and St Thomas NHS Foundation Trust London UK; ^4^ Wellcome EPSRC Centre for Medical Engineering, School of Biomedical Engineering and Imaging Sciences King's College London London UK

**Keywords:** amyloid, amyloidosis, cost, healthcare utilization, transthyretin cardiomyopathy

## Abstract

**Objectives:**

Our primary aim was to evaluate the healthcare resource use associated with the diagnosis of transthyretin amyloidosis cardiomyopathy. Second, we aim to assess the effect of the number of diagnostic tests and clinical contact points on the total time and costs between symptom onset and diagnosis defining a quantitative hypothetical optimized diagnostic pathway.

**Setting:**

Clinical and cost data were collected from patients presenting between 2010 and 2018 in a tertiary referral institution in South London involving two participating hospitals.

**Participants:**

Thirty‐eight adult patients with a definite diagnosis of transthyretin amyloidosis cardiomyopathy were included, mostly male (n = 28, 74%) and of African‐Caribbean descent (n = 23, 64%). We excluded patients without a confirmed transthyretin amyloidosis cardiomyopathy or those on inotersen, patisiran, or diflunisal at point of referral.

**Primary and secondary outcome measures:**

The average time between first presentation and final diagnosis, and the cost per patient per month. By comparing to a more optimal clinical pathway towards diagnosis, we considered what could be the theoretical gain in terms of time to diagnosis and financial savings.

**Results:**

The average time between first presentation and final diagnosis was 2.74 years. The average cost per patient per month was higher with progressive heart failure symptoms. A hypothetical optimal pathway reduces time to diagnosis of 1.65 to 1.74 years per patient. The potential financial savings are estimated within the range of £3000 to £4800 per patient.

**Conclusions:**

Patients diagnosed with transthyretin amyloidosis cardiomyopathy have substantial healthcare resource utilization and costs starting from symptom onset. Higher costs were observed with progression in symptoms and appear linked to a delayed diagnosis. The number of additional diagnostic tests and clinical contact points may contribute to this and could represent a path to explore further for important health and cost savings, with more efficient pathways for these patients to be managed.

## 1 INTRODUCTION

Amyloidosis is a family of diseases, characterized by the pathological deposition of misfolded, insoluble fibril proteins.^1^

Transthyretin amyloidosis (ATTR) exists as two subtypes ‐ age related, also known as wild type TTR (ATTRwt), and an inherited form, also known variant ATTR (ATTRv), which differ in age of onset and disease penetrance.^1^, ^2^ Deposition of the misfolded proteins in both cases can be multisystemic with extracardiac involvement in the central or peripheral nerves, renal and gastrointestinal systems, with symptoms in these affected areas often predating the cardiac manifestations.^3^ This considerable variability in clinical presentations related to the disease necessitates a multidisciplinary approach particularly with close contact between cardiology and neurology specialties to reach an early diagnosis and initiate appropriate management.^4^

Myocardial involvement with development of an infiltrative cardiomyopathy is associated with the worse outcomes, and as such, delays in this diagnosis can be detrimental.^5^, ^6^, ^7^ Confounding this further, is evidence suggesting the ATTRwt form of cardiomyopathy is not uncommon, being overlooked or misdiagnosed as other conditions, such as hypertensive heart disease and heart failure with preserved ejection fraction.^5^, ^8^, ^9^ These patients are at additional risk, as they are likely to receive traditional medical therapy that is either ineffective or are accompanied by adverse outcomes in the presence of this overarching diagnosis.^10^ Emerging, targeted therapies hold promise for improving outcomes in those individuals in whom this condition is correctly and timely identified.^11^, ^12^

The NHS National Amyloidosis Centre (NAC) is commissioned in the UK for the comprehensive evaluation and management advice for the national caseload of patients with amyloidosis.^13^, ^14^ They have provided a useful overview that describes the natural history and outcomes of ATTR cardiomyopathy in the UK.^15^, ^16^

Although there is increased awareness for the condition, due to the increased availability of advanced non‐invasive imaging techniques, the number of new diagnoses is rapidly expanding and it will be challenging for the NAC alone to manage the growing volume of cases.^15^ Compounded by the observation that a significant proportion of patients have multiple admissions and investigations prior to their diagnosis, it is paramount if emerging therapies are on the horizon, that we ensure timely, equitable access for as many patients as possible.

Reconfiguring the care of these patients and collaboration through a network of hub and spoke centers to manage the variable stages of disease progression, may offer a prudent avenue for the NAC to manage these patients. However, gaps still remain discerning the clinical journey towards diagnosis within centers that manage these patients on a day‐to‐day basis, and therefore the associated healthcare utilization and economic burden. A closer review of these aspects, particularly in centers able to deliver long‐term or complex specialized care in addition to urgent and ambulatory services will further help our understanding and assist in restructuring clinically relevant pathways. This could ultimately help minimize delays to diagnosis, enable more streamlined input via the NAC and importantly, improve patient‐centered outcomes for this life‐threatening disease.

## 2 OBJECTIVES

The objectives of this study were 2‐fold. First, we aimed to understand the healthcare resource use and the attributed costs associated with ATTR cardiomyopathy patients at our center, by mapping and describing their clinical journey pre‐ and post‐diagnosis. Second, we sought to estimate the mean pre‐diagnosis cost per patient and explore the hypothesis that a theoretical optimal pathway would have a direct impact on delayed diagnosis and cost accrued pre‐diagnosis. To test this hypothesis, we conducted quantitative analysis of the diagnostic pathway using real‐world data.

## 3 METHODS

### 3.1 Design and context

This is a retrospective, cross‐sectional, descriptive study within the King's Health Partners (KHP), an academic health science center based in London. This was predominantly focused on patients assessed at Guy's and St Thomas' hospitals (GSTT), and any subsequent healthcare resource use at King's College Hospital (KCH). We first registered a retrospective audit on both sites that was approved and conducted to evaluate patients seen with a diagnosis of amyloidosis, alongside their investigation and referral process to the NAC.

Clinical information of patients with ATTRwt or ATTRv with cardiac involvement, diagnosed by GSTT (all of whom were referred to the NAC where the diagnosis was confirmed) between 2010 and 2018 were retrospectively evaluated. Diagnosis was based on the 2019 expert consensus recommendations for the suspicion and diagnosis of ATTR cardiac amyloidosis and included Electrocardiogram (ECG), plasma biomarkers, echocardiography, cardiac Magnetic Resonance Imaging (cMRI) and screening for monoclonal proteins.^17^ Use of radiotracers was also included, and this was done exclusively at the NAC. Invasive methods included endomyocardial, fat, bladder, rectal, bone marrow, nerve, or salivary gland biopsy with histological testing to confirm the preliminary diagnosis. This was followed by characterization of the amyloid type which always occurred at the NAC.

### 3.2 Patient and public involvement

No patient/public involvement.

### 3.3 Inclusion criteria

Patients had to meet all the following criteria to be eligible for inclusion in this study:

1. Have a definitive diagnosis of ATTR cardiomyopathy
2. Adult patients (aged 18 years or older)
3. Diagnosed between January 2010 and December 2018

**FIGURE 1:**
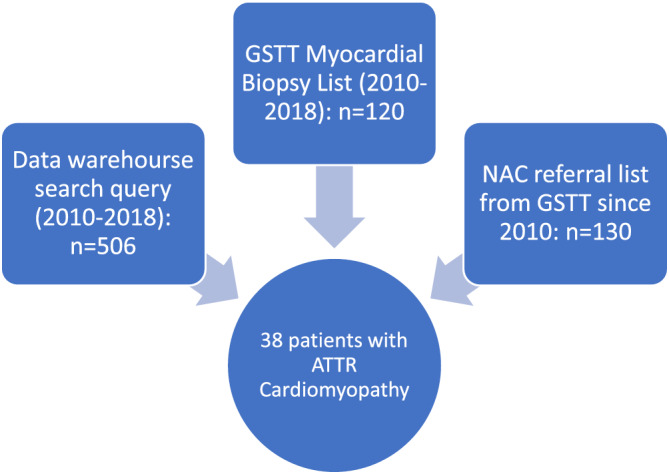
Flow chart summarizing data sources examined and extracted to determine number of transthyretin amyloidosis (ATTR) cardiomyopathy patients. All hospital attendances at Guy's and St Thomas' hospitals (GSTT) are fully coded by the clinical coding team, and entered a data warehouse. This was accessed by our data analyst and any patient coded between 2010 and 2018 with an amyloidosis code were identified. ATTR cardiomyopathy status was determined by examination of clinical notes, referral letters to the National Amyloid Centre (NAC) and biopsies at GSTT. Myocardial biopsy list was provided by the Cardiovascular Quality Improvement and Patient Safety Manager who maintained a database of all cardiac biopsies between 2010 and 2018. Every patient was reviewed using their unique identifier within the electronic health record system to identify the final diagnosis. NAC provided their available referral list from GSTT since 2010. Those with ATTR cardiomyopathy were extracted and cross‐checked in the data warehouse search and biopsy lists to confirm that all with ATTR cardiomyopathy were referred to the NAC

Data sources examined at GSTT are summarized in Figure 1. ATTR cardiomyopathy status was determined by examination of clinical notes, referral letters to NAC and biopsies by one clinician, and a second clinician for data source verification.

We extracted data on all Accident and Emergency (A&E), inpatient, outpatient, and community heart failure contacts through the relevant information systems at GSTT. Some of the cohort (n = 18) also utilized health care resources at KCH. This was manually reviewed via the electronic health record system to identify their activity.

### 3.4 Exclusion criteria

1. Patients on inotersen, patisiran or diflunisal at point of referral to GSTT.
2. Amyloid patients without confirmed ATTR cardiomyopathy.

This resulted in the exclusion of two patients, due to the absence of a confirmed ATTR cardiomyopathy diagnosis. No patients were on inotersen, patisiran or diflunisal at point of referral to GSTT.

### 3.5 Catchment area

Patients within the GSTT catchment area will have received the majority (if not all) of their secondary care at GSTT, and a few also utilized KCH. Initial review of the data suggested that approximately half of the GSTT patients were referred from out of area.

### 3.6 Referrals to the NAC

All confirmed ATTR cardiomyopathy patients were referred to the NAC. When a presumed diagnosis was made from imaging or biopsies, a referral was made to the NAC. In more recent years, patients have also been directly referred to the NAC without cardiac biopsy if there is strong evidence for cardiac amyloid presence on clinical information and imaging alone. Most referrals were made through the cardiology service; however, some patients may have been seen in the hematology service, and referred directly.

### 3.7 Variables

The clinical dataset included demographics, clinical contact points at GSTT during the study period and diagnostic investigations utilized toward diagnosis. The New York Heart Association (NYHA) classification of heart failure was used to describe each patient's symptom severity throughout the pathway. This is widely used in clinical practice, uniform, and an accepted classification of heart failure symptoms.

### 3.8 Mortality data

GSTT sent the list of confirmed ATTR cardiomyopathy patients to the Office for National Statistics (ONS), who provided latest mortality status with date of death (where relevant) for each patient.

### 3.9 Costs

We used NHS reference costs to estimate the aggregated total cost per patient prior and post diagnosis confirmation. This was inclusive of healthcare resource use of outpatient cardiology visits, inpatient admissions, A&E visits, paid‐for community care, with echocardiogram and cMRI as diagnostic tests. We did not consider the cost of biopsy and scintigraphy as we assumed all patients were tested equally at the NAC for diagnosis confirmation.

### 3.10 Economic impact of optimal diagnostic pathway

We hypothesized implementing an optimal pathway may have a positive effect on earlier diagnosis and cost reduction. This analysis consisted of three stages. Firstly, we determined a quantitative definition of an optimal diagnostic pathway using the retrospective data collected. Clinical expertise suggested the minimum number of actions required to determine a diagnosis was an exploratory consultation and investigation upon symptoms onset followed by a confirmatory investigation and consultation before referral to the NAC. We defined a diagnostic cycle as two consultation visits and two tests (or any combination thereof) (see Appendix S1). Consultation options were considered as either outpatient, A&E visits, or inpatient admissions. Echocardiogram and cMRI were considered as diagnostic tests.

We then estimated the effect of the number of cycles on time and cost prior to diagnosis after adjusting for a number of covariates using generalized linear modeling (GLM) (see Appendix S1 for details). Finally, we employed the recycled predictions,^18^ to test the hypothesis of optimal management pre‐diagnosis, assuming optimal management is equivalent to having only one diagnostic cycle. We present the potential average savings of time to diagnosis and total pre‐diagnosis costs. We performed sensitivity analysis to test violation of parametrical assumptions and to quantify uncertainty around point estimates (see Appendix S1 for details). Microsoft Excel, R studio and Stata 15 were used for the analyses.

## 4 RESULTS

### 4.1 Study population

We identified 38 patients with a diagnosis of ATTR cardiomyopathy (n = 20; 53% for ATTRwt, and n = 18; 47% for ATTRv). The mean age at diagnosis was 77 ± 8 years and most patients were male (n = 28, 74%), and of African‐Caribbean descent (n = 23, 64%). The pathogenic variant, V122I also known as p.V142l,^19^ was found in 15 (94%) patients with ATTRv, all of whom were of African‐Caribbean descent. One individual with ATTRv who identified as White‐British was affected by the V30M mutation, also referred to as p.V50M.^20^ The majority of patients with ATTR cardiomyopathy assessed at GSTT were via referral (n = 28, 76%) from primary or secondary care rather than from acute admissions to the trust. The average time between first presentation and final diagnosis was 2.74 years.

### 4.2 Outcome

At time of symptom onset, most had mild heart failure symptoms (57%). Those who were asymptomatic or had mild heart failure symptoms, had on average, a longer period of time under the care of GSTT cardiovascular service prior to a diagnosis. Sixty percentage of patients died at the end of the study period (n = 22), and the mortality rate was similar between the groups (61% for ATTRv; 60% for ATTRwt). Baseline characteristics and outcome are summarized in Table 1.

**TABLE 1:** Clinical data of cohort under study

	Total cohort	ATTRwt	ATTRv
Demographics			
Male, n (%)	28 (74%)	17 (85%)	11 (61%)
Female, n (%)	10 (26%)	3 (15%)	7 (39%)
Age, years	77 ± 7.7	80 ± 6.6	74 ± 7.8
Ethnicity: African‐Caribbean, n (%)	23 (61%)	7 (35%)	16 (89%)
Route of first contact, n (%)			
Referral	29 (76%)	15 (75%)	14 (78%)
NYHA class at symptom onset, n (%)			
I	7 (19%)	3 (15%)	4 (23%)
II	21 (57%)	9 (45%)	12 (71%)
III	5 (14%)	4 (20%)	1 (6%)
IV	1 (3%)	1 (5%)	0
NYHA class closest to diagnosis, n (%)			
I	5 (13%)	4 (20%)	1 (6%)
II	15 (41%)	5 (25%)	10 (59%)
III	10 (27%)	8 (40%)	2 (12%)
IV	6 (16%)	3 (15%)	3 (17%)
Investigations			
cMRI prior to diagnosis, n (%)	32 (84%)	16 (80%)	15 (83%)
Biopsy	28 (73%)	15 (75%)	12 (67%)
Medication use			
ACE I or ARB	29 (76%)	15 (75%)	14 (78%)
B Blocker	31 (82%)	17 (85%)	14 (78%)
Diuretic	34 (89%)	17 (85%)	17 (94%)
Mortality			
	23 (61%)	12 (60%)	11 (61%)

*Note*: The data is presented as mean ± SD, n (%).

Abbreviations: ACE I, angiotensin converting enzyme inhibitor; ARB, angiotensin receptor blocker; ATTRv, variant ATTR; ATTRwt, wild type ATTR; B Blocker, beta blocker; cMRI, cardiac magnetic resonance imaging; NYHA, New York Heart Association classification of heart failure.

### 4.3 Costs

Four patients were missing symptom onset date which were dealt with in the costing analysis by using the first known cardiac‐related activity for each of those patients. When we calculated the total costs pre‐diagnosis, we excluded the four patients without symptom onset date as this was required for each section of this calculation. Only one patient was excluded from the calculation of total costs post diagnosis, as there was insufficient information on their healthcare resource use during the study period.

Tables 2 and 3 summarize the costs from symptom onset to diagnosis and from diagnosis to study exit date. The average cost per patient per month was higher with progressive heart failure symptoms, and driven mostly by the inpatient admission costs. Heart failure community nurses' costs were notably higher from diagnosis to study exit period, presumably relating to more heart failure symptoms as the disease progresses.

**TABLE 2:** Costs (£) from symptom onset to diagnosis and breakdown of components per month per patient

Costs from symptom onset to diagnosis					
NYHA group (at symptom onset)	1	2	3	4	Total
Number of patients	7	21	5	1	34
Total days (onset to diagnosis)	5871	17 167	2034	194	25 226
Average days (onset to diagnosis)	839	817	407	194	
Total Cost	£25 009	£188 393	£38 191	£28 960	£280 553
Average cost/patient/month	£130	£334	£571	£4541	

Breakdown of average monthly cost per patient					
Echocardiogram	£4	£9	£11	£57	
New cardiology outpatient	£15	£17	£17	£0	
Follow up cardiology outpatient	£6	£16	£29	£0	
A&E	£8	£25	£13	£66	
Heart failure community nurse	£0	£3	£0	£0	
Inpatient admission	£97	£258	£502	£1169	
Excess bed days	£0	£6	£0	£3249	

Abbreviations: A&E, Accident and Emergency; NYHA, New York Heart Association classification of heart failure.

**TABLE 3:** Costs (£) from diagnosis to study exit date and breakdown of components per month per patient

Costs from diagnosis to study exit						
NYHA group (at diagnosis)	1	2	3	4	Unknown	Total
Number of patients	6	14	10	6	1	37
Total days (diagnosis to study exit)	3941	10 776	4575	5451	342	25 085
Average days (diagnosis to study exit)	657	770	458	909	342	
Total Cost	£11 883	£122 288	£86 502	£111 262	£17 120	£349 055
Average cost/patient/month	£92	£345	£575	£621	£1523	

Breakdown of average monthly cost per patient						
Echocardiogram	£3	£10	£4	£7	£22	
New cardiology outpatient	£7	£16	£12	£4	£20	
Follow up cardiology outpatient	£16	£38	£36	£33	£99	
A&E	£5	£30	£35	£27	£56	
Heart failure community nurse	£3	£13	£6	£19	£0	
Inpatient admission	£58	£210	£446	£354	£1326	
Excess bed days	£0	£27	£36	£176	£0	

Abbreviations: A&E, Accident and Emergency; NYHA, New York Heart Association classification of heart failure.

### 4.4 Optimal pathway analysis

From the data (n = 38), the average number of diagnostic cycles was 2.13 (SE = 0.21), in a range between 1 and 6.75. The unadjusted mean of time to diagnosis and total costs was 2.74 (SE = 0.44) years and £8607 (SE = 1079.86), respectively. The observed distribution of time to diagnosis and costs were heavily right‐skewed. The results of link and distribution tests for the GLM specification are described in Appendix S1. Table 4 shows the results of the optimal pathway analysis for time to diagnosis and total costs.

**TABLE 4:** Optimal pathway analysis for time to diagnosis (years) and mean total costs pre‐diagnosis (£)

Estimation method	Mean time to diagnosis	Mean total costs pre‐diagnosis
Raw data	2.74 years (SE = 0.44)	£8607 (SE = 1079.86)
GLM	2.74 years (SE = 0.38)	£ 8927 (SE = 1020.31)
GLM‐based optimal	1.01 years (SE = 0.11)	£4193 (SE = 393.52)
GLM‐based savings	1.74 years (SE = 0 .34)	£4734 (SE = 896.68)
Bootstrap + OLS (simulation)	2.73 (SD = 0.43)	£8619 (SD = 1054.82)
Simulation‐based optimal	1.07 (SD = 0.25)	£5533 (SD = 1383.06)
Simulation‐based savings	1.65 (SD = 0.38)	£3087 (SD = 961.82)

Abbreviations: GLM, generalized linear modeling; OLS, ordinary least squares.

#### 4.4.1 Time to diagnosis

The GLM in the analysis of time to diagnosis provided the same average estimate as the observed data while reducing the uncertainty around this point estimate. The implementation of a hypothetical optimal diagnostic pathway generates a mean time to diagnosis of 1.01 years (SE = 0.11). This represents a potential reduction in time to diagnosis of 1.82 years (SE = 0.34). Bootstrap‐based sensitivity analysis provided an adjusted and optimal mean time to diagnosis of 2.73 years (SD = 0.43) and 1.07 years (SD = 0.25), respectively. This difference yields potential reduction in time to diagnosis of 1.65 years (SD = 0.38).

#### 4.4.2 Total cost prior to diagnosis

The GLM estimate for total costs was £ 8927 (SE = 1020.31). Implementing an optimal pathway generates mean total costs of £4193 (SE = 393.52). This number represents potential savings per patient of £4734 (SE = 896.68) when compared to the adjusted mean. Sensitivity analysis results suggest the mean adjusted estimate for total cost before diagnosis is £8619 (SD = 1054.82); whereas the mean total costs when managing optimally £5533 (SD = 1383.06). This analysis indicates potential savings of £3087 (SD = 961.82).

## 5 DISCUSSION

This study is the first UK based study to examine the relationship between healthcare utilization throughout the patient journey with ATTR cardiomyopathy and the associated economic burden occurring within a local institution outside the NAC.

The predominant route of contact with patients seen at our trust was via referral; a significant contribution from primary care for further specialist review of presumed cardiac symptoms. There was also a contribution from other “spoke” secondary care hospitals, due to the nature of our trust being a high‐volume center with regards to both cMRI and invasive cardiac biopsy. The ATTRv subgroup was predominantly African‐Caribbean, and in keeping with the literature, this was due to the V122I mutation.^5^, ^19^ Evidence suggests a strong association between carrier status for this mutation and subsequent development of heart failure.^5^, ^21^ This means GSTT is well placed not only to provide specialist investigation and care for those affected by its cardiac consequences, but by serving a large African‐Caribbean population, we are likely to observe a significant proportion of the healthcare resource use related to this condition. Supplementary to this, and indeed more reflective of complete burden, would be an estimation of these same patients' use of primary and social care in relation to this condition.

Patients with ATTR cardiomyopathy on average utilized increasing health care, as symptoms worsened. Costs accordingly rose in step‐wise fashion, per patient per month from symptom onset: £130 with NYHA class 1 patients to £571 with NYHA class 3 patients; and likewise, post diagnosis: from £92 to £575. This is consistent with evidence that emphasizes congestive heart failure is associated with substantial healthcare utilization, and this is driven mostly by costly hospitalizations, as was seen in this study.^22^, ^23^

From diagnosis, there was additional usage of the community heart failure services, and this was partly the source for the higher overall costs following a diagnosis of ATTR cardiomyopathy. This is consistent with the disease profile over time, that this condition is progressive and debilitating, necessitating increased supportive care and careful titration of heart failure medical therapy.^5^, ^7^, ^21^ Given that traditional medical therapy in the form of beta blockers and ace‐inhibitors are sometimes poorly tolerated in patients with ATTR cardiomyopathy, community heart failure nurses are well placed to monitor patients in between local services and the NAC, and to potentially prevent treatment‐related setbacks. Nationwide, it is recognized that community heart failure nurses contribute to a substantial reduction in hospital admissions related to heart failure, and the associated overall costs per patient.^24^, ^25^ This may suggest, that in areas where this service has already been decommissioned, one might anticipate a considerable upsurge in hospital admissions and its total costs associated with ATTR cardiomyopathy.

Results from a UK‐based study by Lane et al,^15^ utilizing data from 1034 patients at the NAC suggested large delays between symptom onset and diagnosis (>4 years in 40% of patients), particularly with the ATTRwt subtype. In addition, the authors found a significant association between delayed access and poor health‐related quality of life. Our analysis suggests that a hypothetical optimal pathway would have a considerable impact in terms of time to diagnosis and total costs before diagnosis. The implementation of such a pathway generates reductions in time to diagnosis of 1.65 to 1.74 years per patient. The potential financial savings are estimated within the range of £3000 to £4800 per patient. Alongside the data published by Lane et al,^15^ our analysis provides some indication of a potential time‐ and cost‐saving strategy to adequately identify patients with ATTR cardiomyopathy. Nonetheless, our study findings are to be interpreted cautiously as they are not intended to inform the precise clinical decisions to be taken to manage patients optimally. Further clinical and health system research is needed to identify the factors causing delayed diagnosis, and subsequently to implement actions to address them.

## 6 LIMITATIONS

The authors acknowledge limitations to this study. The sample size is considerably small, consisting of 38 heterogenous patients and therefore significant relationships could not be deduced between the associations described from our findings. For three patients prior to diagnosis and one patient post‐diagnosis, we did not have complete data regarding their healthcare utilization. The prospective observational study by the NAC of patients referred to them with a final diagnosis ATTR cardiomyopathy, found that on average, in 534 patients, there was a diagnostic delay of 3.35 years from first presentation.^15^ Whilst the diagnostic delay is slightly longer than our average of 2.74 years, the NAC data incorporates the entire UK caseload of referrals; our study underlines the hospital service usage within a tertiary specialist center, serving adjacent primary care and local secondary care services. There was also higher resource usage reported by the NAC, however they evaluated all hospital service usage, including other medical specialties and surgical services. Our study was focused on cardiac‐related healthcare use given the diagnosis was heavily influenced by investigations predominantly carried out within our cardiology services. It was also limited to service provision within 2 NHS trusts, making it possible for patients to have had additional activity elsewhere, including primary and social care usage.

We acknowledge our definition of optimality may be an oversimplification of a rather complex diagnostic journey. Our analysis is not intended to provide clinical recommendations nor to inform on the value for money of specific diagnostic algorithms. Instead, our study explores the potential benefits and optimistic impact of a streamlined pathway that minimizes healthcare resource utilization. Therefore, the results stemming from our findings are only illustrative. Research on the implications of achieving an earlier diagnosis, including costs and cost‐effectiveness of novel diagnostic algorithms and therapeutics, is more likely to determine the overall effect within the entire clinical pathway.

## 7 CONCLUSIONS

ATTR cardiomyopathy is associated with increased healthcare resource utilization in conjunction with worsening heart failure symptoms. Inpatient admissions and excess bed days account for a significant proportion of this use and overall costs, with a notable contribution from engagement with community heart failure teams. By review of the current patient clinical journey, we estimated that a hypothetical optimal pathway could result in considerable time savings to diagnosis of 1.65 to 1.74 years, and total costs pre‐diagnosis between £3000 and £4800 per patient. In order to appreciate this, it requires a high index of suspicion in “at‐risk” individuals who present mostly within primary or secondary care, with selective diagnostic tools available at highly specialized hubs such as our center, to elucidate the etiology early, manage progressive disease and enable timely, efficient links with the NAC. There is room for further research incorporating primary and social care resource use, but also within data from a national registry to confirm our hypothesis.

## FUNDING

This study was funded by Pfizer UK. The views expressed are those of the author(s) and not necessarily those of the Pfizer UK. No award/grant number was associated with this funding.

## CONFLICT OF INTEREST

Professor Gerry Carr‐White has received research funding from Pfizer UK. The remaining authors on this manuscript have no financial or other relationships to disclose.

## AUTHOR CONTRIBUTIONS

Conceptualization: Clint Asher, Gherardo Finocchiaro, Andrew Guilder, Gerry Carr‐White, Yael Rodríguez‐Guadarrama.

Data Curation: Clint Asher, Andrew Guilder.

Formal Analysis: Clint Asher, Andrew Guilder, Yael Rodríguez‐Guadarrama.

Funding Acquisition: Gerry Carr‐White.

Investigation: Clint Asher, Andrew Guilder, Yael Rodríguez‐Guadarrama.

Methodology: Clint Asher, Gherardo Finocchiaro, Andrew Guilder, Gerry Carr‐White, Yael Rodríguez‐Guadarrama.

Project Administration: Gerry Carr‐White.

Resources: Andrew Guilder, Gerry Carr‐White, Yael Rodríguez‐Guadarrama.

Software: Andrew Guilder, Yael Rodríguez‐Guadarrama.

Supervision: Gerry Carr‐White, Yael Rodríguez‐Guadarrama.

Validation: Andrew Guilder, Gherardo Finocchiaro, Gerry Carr‐White, Yael Rodríguez‐Guadarrama.

Writing – Original Draft: Clint Asher, Gherardo Finocchiaro, Andrew Guilder, Gerry Carr‐White, Yael Rodríguez‐Guadarrama.

Writing – Review & Editing: Clint Asher, Gherardo Finocchiaro, Andrew Guilder, Gerry Carr‐White, Yael Rodríguez‐Guadarrama.

All authors wrote and revised all versions of the manuscript and all authors read and approved the final version of the manuscript.

The authors had full access to all the data in the study and take complete responsibility for the integrity of the data and the accuracy of the data analysis.

## TRANSPARENCY STATEMENT

The authors confirm this manuscript is an honest, accurate, and transparent account of the study being reported; that there is no omission of any important aspects of the study; and that any discrepancies from the study as planned (and, if relevant, registered) have been explained.

## Supporting information


**Appendix S1.** Supporting information.

## Data Availability

Modeling data are available via Appendix S1.
