# Supplementary material for: Healthcare resource use associated with the diagnosis of transthyretin amyloidosis cardiomyopathy
Source: Health Sci Rep. 2022 Jan 6;5(1):e466. doi: 10.1002/hsr2.466 (PMC8733838; doi:10.1002/hsr2.466)
Supplement: Supplementary file 1 — Appendix S1. Supporting information. [file HSR2-5-e466-s001.pdf]

Electronic Supplementary Information

Healthcare resource use associated with the diagnosis of transthyretin amyloidosis cardiomyopathy

Journal: Health Science Reports

Authors

Clint Asher[1,2], Andrew Guilder[1,3], Gherardo Finocchiaro[2], Gerald Carr-White[1,2*], Yael Rodríguez-Guadarrama[4*]

* joint last authors

Institution

School of Biomedical Engineering and Imaging Sciences, Rayne Institute, King's College London, 4th Floor, Lambeth Wing, St Thomas Hospital, London, UK[1]

Department of Cardiology, Guy's and St Thomas' NHS Foundation Trust, London, UK[2]

Care Redesign Improvement and Innovation System, Guy's and St Thomas NHS Foundation Trust, London, UK[3]

Wellcome EPSRC Centre for Medical Engineering, School of Biomedical Engineering and Imaging Sciences, King's College London, London, UK[4]

Corresponding author

Dr Clint Asher

Email: clint.1.asher@kcl.ac.uk

The analysis of the optimal diagnostic pathway consisted in three stages. Firstly, we determined a quantitative definition of a diagnostic cycle using the retrospective data collected (equation 1).

$$\text{Number of diagnostic cycles} = \text{Number of clinical contact points} + \text{Number of diagnostic studies (equation 1)}$$

An optimal pathway was defined as a single diagnostic cycle consisting of two contact points between patient and clinician as well as two diagnostic tests (equation 2). The rationale of this definition is based upon an assumption of a simplified clinical pathway. This consists of a first consultation upon symptoms onset where an explorative test is performed, then a second consultation and test for confirmation. Consultation options were considered as either outpatient, emergency visits or inpatient admission. Echocardiogram and cardiac MRI were considered as diagnostic tests.

$1 = 2a + 2b$ (equation 2)

Where a is the factor of number of consultations and b is the factor of number of diagnostic tests

Furthermore, we estimated the number of diagnostic cycles for every subject in the dataset using equation 3.

Number of cycles=0.25*Number of clinical contact points + 0.25 *Number of diagnostic studies (equation 3)

The estimated effect of the number of cycles on time and cost prior to diagnosis after adjusting for a number of covariates in two separate models as seen below:

$$\text{Time to diagnosis} = \beta_0 + \beta_1\text{Cycles} + \beta_2 \text{ Local} + \beta_3\text{Sex} + \beta_4\text{Age}^3 + \beta_5\text{Age}^3\ln(\text{Age}) + \beta_6\text{NYHA state} + \mu \ldots (1)$$

$$\text{Total cost pre diagnosis} = \beta_0 + \beta_1\text{Cycles} + \beta_2 \text{ Local} + \beta_3\text{Sex} + \beta_4\text{Age}^3 + \beta_5\text{Age}^3\ln(\text{Age}) + \beta_6\text{NYHA state} + \mu \ldots (2)$$

These analyses used statistical modelling allowing for adjustment of the case-mix. Both models were specified as generalised linear models (GLM). Family distribution and link function selection were informed by the Park test and the Pregibon, Pearson and Hosmer & Lemeshow tests respectively. Specification of continuous variables was guided by fractional polynomials.

Finally, we employed the recycled predictions (Glick, et al. 2014) to test the hypothesis of optimal management pre diagnosis, assuming optimal management is equivalent to having only one diagnostic cycle. Effectively, we

replaced the estimated number of cycles with the assumption that all patients were managed optimally. We present the potential average savings of time to diagnosis and total pre-diagnosis costs.

We performed sensitivity analysis to test violation of parametrical assumptions and to quantify uncertainty around point estimates. This approach consisted on an iterative regression model using bootstrap replicates. An ordinary least squares (OLS) model was chosen in order to avoid the risk of non-convergence due to data structure (Santos Silva and Tenreyro 2010). Uncertainty is reported as the standard deviation of the resulting distribution.

Base case regression analyses results

Time to diagnosis

Link: log, distribution: gamma

	Coef.	Std. Err.	Z	P>z	[95% Conf.	Interval]
Cycles	0.6028528	0.1143277	5.27	0	0.3787747	0.826931
Local	-0.5364679	0.2750471	-1.95	0.051	-1.07555	0.0026145
Age	0.0386437	0.0181208	2.13	0.033	0.0031275	0.0741598
Sex	-0.8479547	0.2931685	-2.89	0.004	-1.422554	-0.273355
LOS	-0.0078356	0.0055442	-1.41	0.158	-0.018702	0.0030308
NYHA state	-0.2618233	0.1937826	-1.35	0.177	-0.6416302	0.1179837
Constant	-2.039853	1.314172	-1.55	0.121	-4.615583	0.5358766

Total cost prior to diagnosis

Link: log, distribution: gamma

	Coef.	Std. Err.	Z	P>z	[95% Conf.	Interval]
Cycles	0.3766135	0.1075682	3.5	0	0.1657836	0.5874434
Local	0.2612903	0.2517859	1.04	0.299	-0.232201	0.7547817
NYHA state	0.4854179	0.2092114	2.32	0.02	0.0753711	0.8954647
LOS	0.0069351	0.0051094	1.36	0.175	-0.0030793	0.0169494
Constant	6.825859	0.5104137	13.37	0	5.825466	7.826251

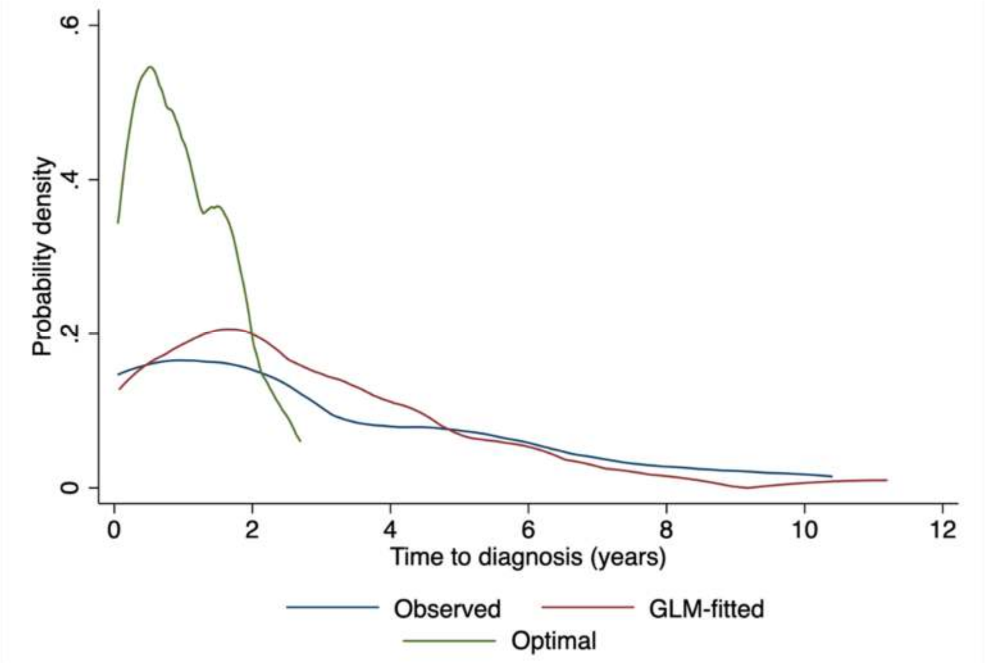

Figure A. Probability densities of observed, generalised linear modelling (GLM)-fitted and optimal time to diagnosis.

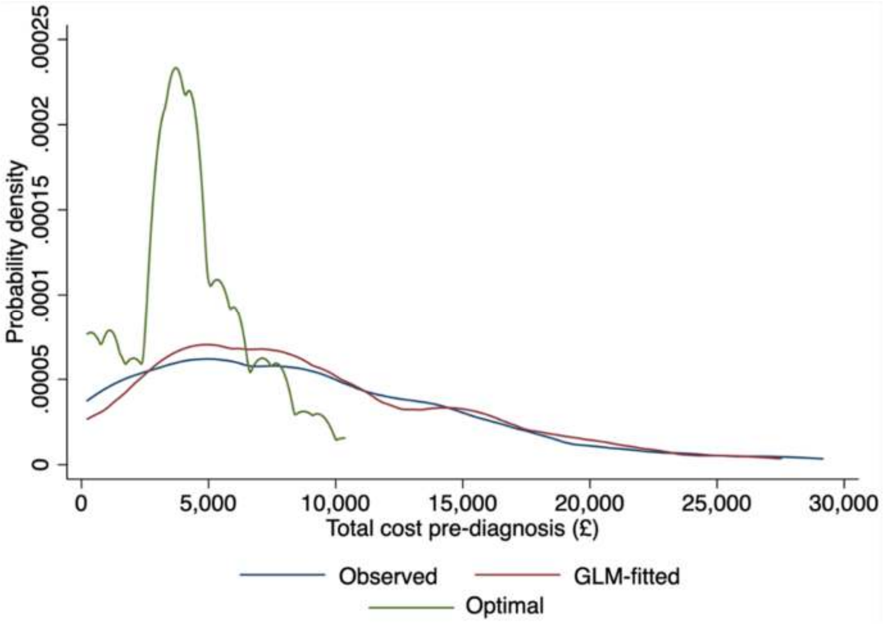

Figure B. A graphical summary of the probability densities corresponding to the observed, generalised linear modelling (GLM)-fitted and optimal values of costs prior to diagnosis.

References

Glick, Henry A., Jalpa A. Doshi, Seema S. Sonnad, and Daniel Polsky. 2014. *Economic Evaluation of Clinical Trials.* Oxford: Oxford University Press.

Santos Silva, J. M. C., and Silvana Tenreyro. 2010. "On the existence of the maximum likelihood estimates in Poisson regression." *Economics Letters* 107 (2): 310-312.
